# Estimating the number of injection drug users in greater Victoria, Canada using capture-recapture methods

**DOI:** 10.1186/1477-7517-11-9

**Published:** 2014-03-03

**Authors:** Yuan Xu, Murray Fyfe, Liz Walker, Laura LE Cowen

**Affiliations:** 1 Department of Mathematics and Statistics, University of Victoria, PO Box 3060 STN CSC, Victoria, BC V8W 3R4, Canada; 2 Vancouver Island Health Authority, #430-1900 Richmond Avenue, Victoria, BC V8R 4R2, Canada

**Keywords:** Injection drug user, Public health, Capture-recapture, Population size

## Abstract

**Background:**

Population size estimation is critical for planning public health programmes for injection drug users. Estimation is difficult, as these populations are considered 'hidden’ or 'hard to reach’. The currently accepted population size estimate for greater Victoria, Canada is between 1,500 and 2,000 individuals, which is dated prior to the year 2000, and is likely an underestimate.

**Methods:**

We used three mark-recapture methods (the Lincoln-Petersen estimator, Huggins' model, and Pledger's model) to estimate population size using cross-sectional survey data collected in 2003 and 2005. Data come from a closed population with two time-ordered samples from the same source. We compare our estimates with the currently accepted estimate that is based on the registry of a Victoria needle exchange.

**Results:**

All methods provided population size estimates that were higher than the currently accepted estimate. Huggins' method produced wider confidence intervals. Point estimates of population size from the three methods ranged from 3,329 to 3,342.

**Conclusions:**

Our estimates will aid health authorities in planning for harm reduction programmes. Repeating the methods as further phases of I-Track data become available will ensure that the population estimates remain up to date.

## Background

Prevalence of HIV and hepatitis C in many injection drug user (IDU) populations is higher than in the general population; the same can be said for the injection drug user population of greater Victoria, British Columbia, Canada (City of Victoria and the 12 other members of the Capital Regional District) where both open and hidden use are known to occur [1]. IDUs are faced with many other challenges to their well-being, and public health authorities are charged with the duty of providing various harm prevention services from basic health care, addictions treatment, and counselling, to harm reduction education. Knowledge of the number of injection drug users within a population would aid both health authorities and community organisations in assessing coverage of existing programmes and in the planning and delivery of a range of public health services.

AIDS Vancouver Island (AVI)'s needle exchange programme was established in 1988, providing clean syringes for IDU residents of Victoria and surrounding areas including the Gulf Islands. The client load of AVI's needle exchange programme [2] was used to produce the only estimate available for the number of IDUs in the Capital Health Region. This estimate published in 2000 was 1,500–2,000 individuals [1]; however, there are no specific details on how this estimate was determined. In 2008, the fixed-site needle exchange location in Victoria was closed, and needle exchange services are now provided on a mobile basis. Other agencies have also started offering clean supplies to IDU clients in Victoria since the client load estimate was generated. It is therefore unknown how reliable the use of the current needle exchange programme registry is for assessing the size of the IDU population in greater Victoria. An accurate estimate of IDUs is vital to the planning of health services for this population.

To track changes in the prevalence of HIV and hepatitis C as well as risk behaviours, the Public Health Agency of Canada in collaboration with regional health authorities developed the national, cross-sectional I-Track survey [3]. Phase I and phase II of the I-Track survey were completed in Victoria in 2003 and 2005, respectively. With only two samples from the I-Track survey (phase I and phase II), a closed population model must be implemented, as three or more samples are required to implement open population models. We use three closed population mark-recapture models to estimate the number of IDUs in greater Victoria, BC and compare the estimates obtained.

### Mark-recapture models

Mark-recapture or capture-recapture models come from the desire to estimate demographic parameters of wildlife populations. Their use in epidemiology is most prevalent from a multi-list standpoint where data from several sources are combined to serve as samples from a population of interest [4]. Multiple data lists are typically collected over the same time frame but from different sources. For example, Hickman et al. studied injection drug use in Brighton, Liverpool, and London from five sources, namely arrest referrals, drug treatment reports, syringe exchange programmes, accident and emergency records, and a community recruitment survey [5]. For two-data source studies, samples may be dependent and there is no means to test for independence unless three data sources are obtained. This is the advantage of time-ordered samples—one can model dependence through the behaviour of the injection drug users (see discussion on trap-happy or trap-shy behaviour). Hook and Regal provide an overview of the use of mark-recapture multi-list methods [6]. In multi-list studies, there is no natural time ordering to the lists; thus, not all wildlife estimation techniques are valid [7]. It is less common to see epidemiological studies that sample the population over time, likely due to the logistics and resources required for such an undertaking (see [8,9] for examples). However, if done, the time ordering of samples offers an opportunity to use different estimation procedures than in multi-list studies.

In wildlife studies, individuals are captured, marked with a unique identifier, and returned to mix back into the population. In subsequent samples, marked individuals are identified (recaptured) and unmarked individuals are given marks before release. Thus, an animal's capture history is recorded and is represented by a sequence of 0's (not captured) and 1's (captured) for each sample occasion. For example, in a two-sample study, an animal with a history of {11} was caught at time 1, tagged, and released back into the population and was recaptured at time 2. In contrast, an animal with a capture history of {10} was caught at time 1, tagged and released, and was not seen again.

In studies of human populations, individuals are contacted (captured), and unique identifiers are obtained (marks). Here unique identifiers could be some combination of a person's date of birth, initials, age, etc. In subsequent samples, individuals are again contacted, and unique identifiers are obtained. Individuals whose identifiers match those from the first sample are considered to be re-sampled (recaptured). Once more, a capture history is developed for each individual in the study. For example, in a two-sample study, an individual with a capture history of {11} was contacted in the first sample, marks were obtained, and the individual was contacted in the second sample. An individual with a capture history of {01} was only contacted in the second sample. For the purposes of this paper, the mark-recapture terminology used in wildlife models will be used to refer to human populations.

The three estimators we implemented were the Lincoln-Petersen estimator [10], a conditional likelihood estimator [11,12], and a maximum likelihood estimator with finite mixtures [13]. The Lincoln-Petersen (LP) estimator (see [10]) is widely used in epidemiological two-sample studies (for example, see [8,9]). Its limitations are largely due to model assumptions, which are similar for the other methods that we explored and are as follows:

1. The population is closed (no births or deaths, immigrations or emigrations).

2. The probability of capture is the same for each individual in the population within a sample.

3. Samples are independent.

4. Marks are not lost.

The other two estimators share these assumptions but provide methods to relax assumption 2.

Assumption 2 leads to the assumption that samples are independent. Chao describes causes for dependent samples, which include behavioural responses (e.g. trap-happy or trap-shy—see discussion) and heterogeneity in capture probabilities [7]. Incorporation of dependence among samples can be done by relaxing assumption 2 [7], implementing methods reviewed by Otis et al. [14].

The LP estimator violates assumption 2 when behaviour and/or heterogeneity affects the probability of capture. One method of dealing with heterogeneity in the data is to incorporate covariates into the estimation procedure. To do so, Huggins introduced a conditional likelihood procedure where capture probabilities can vary according to age, sex, or other factors [11]. Because the covariates for uncaptured individuals are unknown, Huggins constructed a likelihood conditional on the captured individuals so that characteristics of uncaptured individuals are not required [11]. Huggins' method also allows capture probabilities to depend on an individual's prior capture history [11]. The population size is then estimated indirectly using the capture probability estimates.

Another estimation procedure that models capture probabilities dependent on time, behaviour, and/or heterogeneity was proposed by Pledger, introducing finite mixture models to partition the individuals into two or more groups with relatively homogeneous capture probabilities [13]. Pledger's method relaxes assumption 2 but does not condition on captured individuals [13]. Rather, the likelihood models both captured and non-captured individuals, allowing the size of the population (*N*) to be a parameter that is estimated directly. Xu and Cowen detail these three methods [15].

## Methods

### I-track survey

The I-Track survey in Victoria is thoroughly described elsewhere [3]. Briefly, consenting participants were recruited in the downtown core of Victoria through a needle exchange programme run by AVI and at shelter services run by the Victoria Cool Aid Society. Other recruitment attempts were done using posters, flyers, word of mouth, and through contact with Vancouver Island Health Authority staff. Participants were not required to have a residence in Victoria or to have resided in Victoria for any specific period of time. Monetary compensation ($20.00) was provided for answering a questionnaire and providing a blood spot sample. Demographic and risk behaviour statistics resulting from these surveys are reported elsewhere [3]. Phase I completed in November 2003 had 254 participants, while phase II completed in June 2005 had 250 participants.

Eligibility criteria included being at least 15 years of age, being capable of informed consent, having an understanding of English or French, having injected non-therapeutic drugs in the past 6 months, and participation only once per phase. Parental consent was not needed, as it is possible to have mature minor consent in British Columbia.

Survey participants were asked to provide their initials, gender, and birth date (no proof of identification was required for I-Track participation). A computer encryption program used these inputs to create a unique identifier that would be replicated if the same data were entered again in a future phase of the study. This allowed the subjects to be linked between different study phases and preserved anonymity. This identifier (analogous to a unique tag in a wildlife study) is the tool that allows for a mark-recapture study, resulting in the estimation of the number of injection drug users in greater Victoria, BC.

To establish that respondents were injection drug users, subjects were recruited only after an exchange of needles had taken place at the needle exchange. In other locations, screening questions were used (e.g. Where on your body do you inject? Where do you get your rigs? What size needle do you use? When did you last inject?). If during the interview the subjects' responses suggested a lack of familiarity with terms, their eligibility would be questioned.

### Statistical analysis

We discuss the details of the statistical models in the Appendix. We implemented models in Program MARK [16]. Model selection was done by forming a set of plausible models and using Akaike's information criterion corrected for small sample sizes (AICc) to choose a model from among this candidate set [17]. Goodness of fit for closed population models has not yet been resolved [18] (see the Appendix for a discussion). However, we did compare observed with expected counts of each capture history in the form of Pearson chi-square residuals (i.e., *X*^2^ = (observed - expected)^2^/expected) [19].

## Results

Table 1 provides basic demographic characteristics of the two phases of I-Track data.

**Table 1:** Demographics of phases I and II of the I-Track survey

	**Phase I 2003**	**Phase II 2005**
Percent male	73.5	76.0
Percent with high school education or greater	48.4	50.0
Percent aboriginal	20.6	20.9
Average age (years)	34.6	38.8
Average age of first injection (years)	23.0	22.8
HIV prevalence (%)	15.4	12.5
HCV prevalence (%)	68.5	73.8

A thorough discussion of the model selection process for each method is discussed in Xu and Cowen [15]. Briefly, we examined the eight standard closed population models outlined by Otis et al. [14]. These models allow capture probabilities to vary by time, behaviour, and/or heterogeneity. The Lincoln-Petersen estimator is the model where capture probabilities vary by sample time. Of the 254 individuals sampled in phase I and 250 individuals sampled in phase II, there were 19 individuals in both samples. The population size estimate is 3,329 individuals using the Lincoln-Petersen estimator (Table 2). For Huggins' method, both the individual's sex and previous capture history were used as covariates for modelling the heterogeneity in capture probability. We also examined models to see if there was additional group heterogeneity. However, AICc chose the model with constant capture probabilities and no group heterogeneity. Similar results occurred with Pledger's method; there were no time, behaviour, nor heterogeneity responses in the capture probabilities. The model with constant capture probabilities had the lowest AICc value.

**Table 2:** The estimated number of injection drug users in greater Victoria, BC

**Model**	N^	**SE**	**95% confidence interval**
LP	3,329	706	(2,246, 5,078)
H	3,342	709	(2,254, 5,098)
P	3,330	706	(2,246, 5,078)

N^, estimated population size; SE, estimated standard error; LP, Lincoln-Petersen model; H, Huggins' conditional likelihood model; P, Pledger's mixture model.

Table 2 compares the estimation results from all three estimation methods. In terms of the point estimate for population size and the confidence intervals, all three methods produced similar results; however, none of the confidence intervals contains the upper bound estimate of '2,000’ provided by Stajduhar et al. [1]. Further, the estimated standard errors for all methods were also similar.

Pearson chi-square residuals for the model with constant capture probability (Pledger's model) are provided in Table 3. Based on these results, we find no evidence for outliers or concerns with fit of the model. Similar results were seen for residuals of Huggins' model and the LP estimator.

**Table 3:** Observed count, expected count, and Pearson chi-square residual for the model with constant capture probability

**Capture history**	**Observed**	**Expected**	**Residual**
11	19	19.06	0.00
10	235	232.91	0.02
01	231	232.91	0.02

## Discussion

There was some concern that the number of recaptures in our study was lower than expected, resulting in population estimates that were higher than the currently accepted estimate of 1,500–2,000 individuals. The I-Track survey aimed to recruit from a broad spectrum of user groups. Forty percent of the I-Track participants were recruited at locations other than the needle exchange. An IDU population estimate based on the needle exchange programme registry prior to the year 2000 (the 1,500–2,000 estimate) would miss people who were not clients of the needle exchange programme and is therefore likely an underestimate. Our estimate represents approximately 0.9% of the greater Victoria population, whereas the proportion of IDUs is approximately 0.2%–0.9% nationwide [20,21]. However, Victoria has a comparably mild climate that may attract street-involved people from other areas. We therefore argue that it is reasonable for our estimate to be at the upper end of national estimates. Because the national estimate is based on a population survey that covers both urban and rural locations, it is not directly comparable to our estimate.

**Figure 1:**
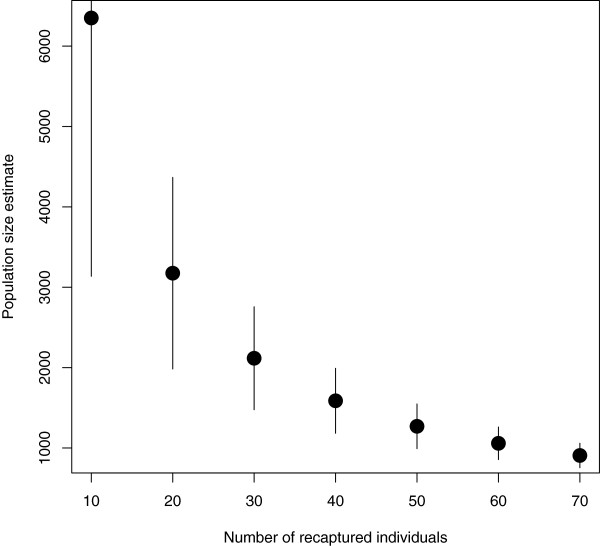
**Estimated population size using the Lincoln-Petersen estimator varying the number of recaptures.** Error bars represent the point estimate ± two estimated standard errors.

A lowered recapture rate could also be the result of a 'trap-shy’ response where individuals from the first survey avoid being captured in the second survey. For the Lincoln-Petersen estimate, this would have resulted in an overestimate of population size [7]. However, as behaviour was modelled in Huggins' and Pledger's models, we would have seen a reduction in the population estimate; this was not the case.To explore this issue further, we varied the number of recaptures in the Lincoln-Petersen estimator to see how this affected the population size estimate (Figure 1). To get a Lincoln-Petersen estimate of around 2,000 individuals, the number of recaptures would have to be at least 32 individuals. Similarly, having 43 recaptures would produce an estimate of around 1,500 individuals.

The closure assumption is likely violated for the I-Track data. Deaths could have occurred between the two I-Track surveys, people could have moved into or out of the region, and initiation or cessation of injection could have occurred between samples. To look at the stability of injection, we define the average number of years of injection as average age minus average age of first injection (Table 1); we find this to be 11.6 and 16.0 years for phases I and II, respectively. The stability of the client groups associated with recruitment sites is unknown and may have had some impact on the closure assumption.

Violation of the closure assumption can result in biased estimates, which increases with increased mobility into and out of the population [22]. Kendall studied the effect of closure violations on closed population models from the viewpoint of individuals in the population being a subset of a superpopulation [23]. For situations where individuals are able to move randomly in and out of the study area throughout the study, Kendall considered each of the survey samples to be random samples from a superpopulation of size *N*^0^[23]. Individuals in the study area are drawn from the superpopulation with probability *τ*_*j*_ and captured with probability *p*_*ij*_ on occasion *j*. The closed population estimators are biased for the group of individuals in the study area on occasion *j*, but unbiased for the superpopulation. Arguably, the superpopulation is of more interest than the number of individuals in the study area at a particular occasion. The superpopulation for our study would be all individuals that entered the study area between 2003 and 2005.

The assumption of homogeneity of capture probabilities is rarely met in epidemiological studies [24]. This can be affected by the behaviour of an individual. For example, in animal studies, an animal that enjoyed the experience of being caught can become 'trap-happy’. Similarly, if an IDU enjoyed the experience of the first I-Track study or was positively impacted by the $20.00 remuneration, the person might have looked for opportunities to participate in the second. On the other hand, if an individual did not have a good experience with the first I-Track survey, the person might avoid the second survey ('trap-shy’). Further, different individuals could have intrinsically different capture probabilities, causing heterogeneity. Otis et al. specified models that incorporated potential sources of variation by modelling capture probabilities as dependent on time, behaviour, and/or heterogeneity [14]. All of the models we used were based on Otis et al.'s work [14]. The conditional model approach modelled capture probabilities dependent on the sex of the individual, thereby having the potential to further reduce heterogeneity. These models cannot account for individuals who have a null probability of being captured. If such individuals exist in the population, then our estimates would be considered conservative.

As mentioned, the assumption of independent samples can be relaxed and modelled through incorporation of behavioural effects or heterogeneity in capture probabilities. This assumption was not likely violated as models that included behaviour or heterogeneity effects were not selected.As no formal identification is required to participate in the study, it is possible for unique identifiers to change from one survey to the next, violating the assumption of no tag loss. This could happen if an individual forgot the information that results in their unique identifier or if an individual's unique identifier changed between survey phases due to unusual cases such as a name being changed (due to marriage for example). If a subject provided different identifiers, it would not be possible to link them. We argue that this would be rare and would result in a reduced number of recaptured individuals producing overestimates of population size (see Figure 1).

When the data from the latest I-Track survey are available, we would like to use an open population Jolly-Seber model to remove this assumption altogether in future work [25,26].

As our estimate is quickly becoming a decade old, further estimates to determine if the population size remained the same over the last 10 years would be beneficial. Moving into an open population framework with more data would also allow us to assess whether the population size has changed over time. Once established, application of this model to future phases of the data would be relatively straightforward.

## Conclusions

For the Vancouver Island Health Authority, our population estimates will be helpful in the planning of services to meet the health care needs of the IDU population. When harm reduction programmes such as fixed-site needle exchanges are implemented to help control the transmission of HIV and hepatitis C, knowing the number of potential clients will aid in programme development.

Local experience in Victoria has demonstrated that when services are insufficient to meet demand, higher risk drug use practices may take place, including needle sharing. These higher risk practices may result in threats to health such as blood-borne pathogen infections, abscesses, and overdoses.

Improved estimates of the population size will assist in securing resources required to meet service demands and planning the mix of services that may best meet these needs. This could include adjustments to number and types of locations providing harm reduction services, hours of operation, and numbers of staff. Improved estimates will also better enable an assessment of the impact of programmes and policies for this population.

## Appendix

### Statistical model details

As the I-Track data have a natural time ordering, we were not limited to multi-list models. We were able to relax the constant probability assumption using Huggins' model with capture probabilities dependent on the covariate sex [10]. For our model, the capture probabilities were modelled using a linear logistic formulation as

$$\text{log}\;\left(\frac{P_{\mathrm{ij}}}{1\;-\;P_{\mathrm{ij}}}\right)\;=\;\beta_{0}\;+\;\beta_{1}{\text{sex}}_{i}\;+\;\beta_{2}z_{\mathrm{ij}}$$

where *p*_*ij*_ denotes the probability that individual *i* is captured at occasion *j*, sex_*i*_ is an indicator variable for the sex of individual *i*, and *z*_*ij*_ is equal to 1 if individual *i* was captured before occasion *j* and 0 otherwise. Thus, covariates for sex and previous capture history (behaviour of the individual) were introduced into the model.

Using Pledger's method, capture probabilities were modelled dependent on time, behaviour, and/or heterogeneity. The capture probabilities were modelled with a linear logistic formulation as

$$\begin{array}{ll} \text{log}\;\left(\frac{\theta_{\text{jba}}}{1\;-\;\theta_{\text{jba}}}\right)\;= & \;\mu \;+\;\tau_{j}\;+\;\beta_{b}\;+\;{\left(\tau ,\beta \right)}_{\mathrm{jb}}\\ & \;+\;{\left(\beta ,\eta \right)}_{\mathrm{ba}}\;+\;{\left(\tau ,\beta ,\eta \right)}_{\text{jba}} \end{array}$$

where *θ*_*jba*_ is the probability of capture for individual *i* at occasion *j* with behaviour *b* in group *a*; *b* = *b*_*ij*_ is equal to 1 if individual *i* was not caught before occasion *j* and 2 otherwise; *τ*_*j*_ is the effect of time for occasion *j*; *β*_*b*_ is the effect of behaviour for an individual with behaviour *b*; *η*_*a*_ is the effect of heterogeneity for an individual in group *a* = 1, 2,…, *A* with probability *π*_1_, *π*_2_,…, *π*_*A*_; and *μ* is a constant unknown parameter.

### Goodness of fit

Goodness of fit in closed population models is problematic and is still a current statistical issue [18]. One of the main problems is that when heterogeneity is considered in the capture probabilities, there is an infinite number of saturated models due to the fact that individuals that are not captured cannot have their covariates measured; in other words, the saturated model is not uniquely determined due to the missing covariates. This is a problem for a formal goodness-of-fit test based on the deviance, which requires a uniquely specified saturated model. A goodness-of-fit test based on the conditional distribution of the observed data does not suffer from this problem. However, Link pointed out that very different capture probability models can give rise to an identical conditional distribution [27], rendering any goodness-of-fit test based on the conditional distribution powerless in distinguishing these capture probability models.

## Competing interests

The authors declare that they have no competing interests.

## Authors' contributions

YX and LLEC developed the study design, analysed the data, and wrote the first draft of the manuscript. LW and MF provided insight into the interpretation of the results and edited the manuscript. All authors contributed to and have approved the final manuscript.

## References

[B1] Stajduhar K Poffenroth L Wong E Archibald C Sutherland D Rekart M Missed opportunities: injection drug use and HIV/AIDS in Victoria, Canada Int J Drug Policy 2004 15 171 181 10.1016/j.drugpo.2004.01.001

[B2] Stajduhar KI Poffenroth L Wong E Missed Opportunities: Putting a Face on Injection Drug Use and HIV/AIDS in the Capital Health Region 2002 Vancouver, BC: Centre for Health Evaluation and Outcome Sciences (CHÉOS) Scientific Monograph Monograph 10

[B3] Vancouver Island Health Authority I-Track survey: enhanced surveillance of risk behaviours and prevalence of HIV and hepatitis C among people who inject drugs http://www.viha.ca/NR/rdonlyres/5E14E205-5398-4267-AA61-34F9D671F22B/0/Final_ITRACK_Report_Victoria_20060605.pdf

[B4] Domingo-Salvany A Hartnoll RL Maguire A Brugal MT Albertin PA Caylà JA Casabona J Suelves JM Analytical considerations in the use of capture-recapture to estimate prevalence: case studies of the estimation of opiate use in the metropolitan area of Barcelona, Spain Am J Epidemiol 1998 148 732 740 10.1093/oxfordjournals.aje.a009694 9786228

[B5] Hickman M Higgins V Hope V Bellis M Tilling K Walker A Henry J Injecting drug use in Brighton, Liverpool, and London: best estimates of prevalence and coverage of public health indicators J Epidemiol Community Health 2004 58 766 771 10.1136/jech.2003.015164 15310803 PMC1732885

[B6] Hook EB Regal RR Capture-recapture methods in epidemiology: methods and limitations Epidemiol Rev 1995 17 243 264 8654510 10.1093/oxfordjournals.epirev.a036192

[B7] Chao A An overview of closed population capture-recapture models J Agric Biol Environ Stat 2001 6 158 175 10.1198/108571101750524670

[B8] Khan SI Bhuiy A Uddin ASMJ Application of the capture-recapture method for estimating number of mobile male sex workers in a port city of Bangladesh J Health Popul Nutr 2004 22 19 26 15190808

[B9] Minh TT Nhan DT West GR Durant TM Jenkins RA Huong PT Valdiserri RO Sex workers in Vietnam: how many, how risky? AIDS Educ Prev 2004 16 389 404 10.1521/aeap.16.5.389.48740 15491951

[B10] Seber GAF The Estimation of Animal Abundance 1982 2 London: Griffin

[B11] Huggins RM On the statistical analysis of capture experiments Biometrika 1989 76 133 140 10.1093/biomet/76.1.133

[B12] Huggins RM Some practical aspects of a conditional likelihood approach to capture experiments Biometrics 1991 47 725 732 10.2307/2532158

[B13] Pledger S Unified maximum likelihood estimates for closed capture-recapture models using mixtures Biometrics 2000 56 434 442 10.1111/j.0006-341X.2000.00434.x 10877301

[B14] Otis DL Burnham KP White GC Anderson D Statistical inference from capture data on closed animal populations Wildl Monogr 1978 62 1 135

[B15] Xu Y Cowen L Use of closed population models to estimate the number of injection drug users in Victoria, B.C Mathematics and Statistics Technical Report #DMS-865-IR University of Victoria, Victoria, B.C: Department of Mathematics and Statistics https://dspace.library.uvic.ca:8443//handle/1828/3361

[B16] White GC Burnham KP Program MARK: survival estimation from populations of marked animals Bird Stud 1999 46 Supplement 120 138

[B17] Burnham KP Anderson DR Model Selection and Multimodel Inference: A Practical Information-Theoretical Approach 2002 2 New York: Springer

[B18] Lukacs P Cooch E, White G Closed population capture-recapture models Program MARK: A Gentle Introduction 2011 9 1 38 http://www.phidot.org/software/mark/docs/book/

[B19] Williams BK Nichols JD Conroy MJ Analysis and Management of Animal Populations. Modeling Estimation, and Decision Making 2002 San Diego: Academic

[B20] Health Canada Canadian alcohol and drug use monitoring survey Drug and Alcohol Use Statistics 2011 http://www.hc-sc.gc.ca/hc-ps/drugs-drogues/stat/_2011/summary-sommaire-eng.php

[B21] Canadian Centre on Substance Abuse (CCSA) Injection drug users overview Canadian Centre on Substance Abuse 2011 http://www.ccsa.ca/Eng/Pages/default.aspx. accessed in August 2011

[B22] Larson A Bammer G Why? Who? How? Estimating numbers of illicit drug users: lessons from a case study from the Australian Capital Territory Aust N Z J Public Health 1996 20 493 499 10.1111/j.1467-842X.1996.tb01628.x 8987219

[B23] Kendall WL Robustness of closed capture-recapture methods to violations of the closure assumption Ecology 1999 80 2517 2525

[B24] Stephen C Capture-recapture methods in epidemiological studies Infect Control Hosp Epidemiol 1996 17 262 266 10.2307/30141030 8935735

[B25] Jolly GM Explicit estimates from capture-recapture data with both death and immigration-stochastic model Biometrika 1965 52 225 247 14341276

[B26] Seber GAF A note on the multiple recapture census Biometrika 1965 52 249 259 14341277

[B27] Link WA Nonidentifiability of population size from capture-recapture data with heterogeneous detection probabilities Biometrics 2003 59 1123 1130 10.1111/j.0006-341X.2003.00129.x 14969493

